# Supplementary material for: Towards unified AI-driven fracture mechanics: the extended deep energy method (XDEM)
Source: Nat Commun. 2026 Aug 15;17:8492. doi: 10.1038/s41467-026-76748-1 (PMC13484471; doi:10.1038/s41467-026-76748-1)
Supplement: Supplementary file 3 [file 41467_2026_76748_MOESM3_ESM.zip › pic/Method/williams/crack_u2_displacement_right_williams.pdf]

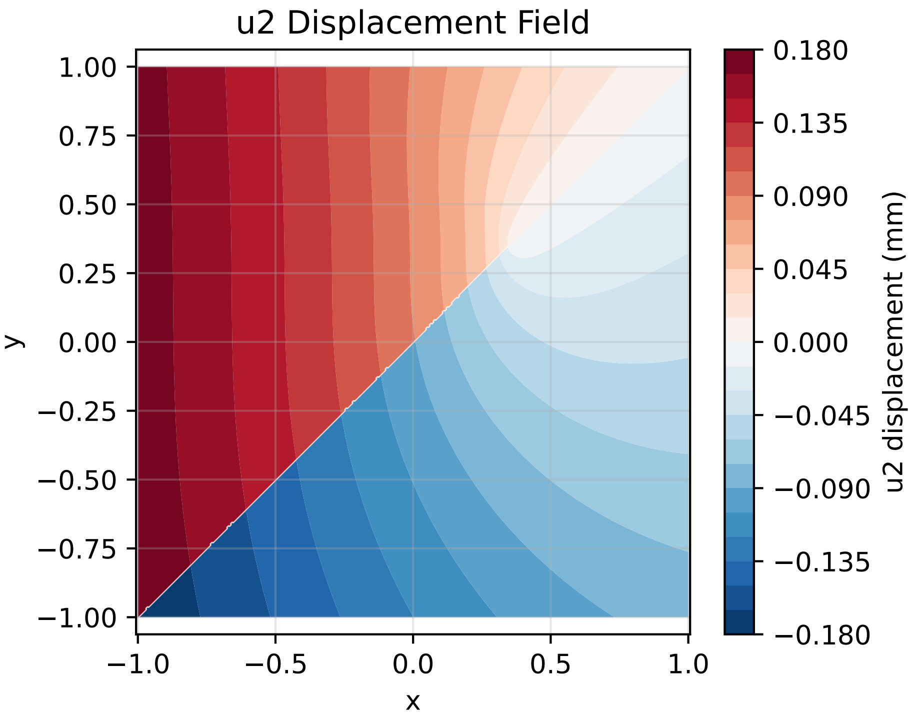
u2 Displacement Field
y
x
1.00
0.75
0.50
0.25
0.00
−0.25
−0.50
−0.75
−1.00
−1.0
−0.5
0.0
0.5
1.0
0.180
0.135
0.090
0.045
0.000
−0.045
−0.090
−0.135
−0.180
u2 displacement (mm)
